# Molecularly Imprinted Polymers Using Yeast as a Supporting Substrate

**DOI:** 10.3390/molecules28207103

**Published:** 2023-10-15

**Authors:** Zhigang Wang, Zhuangzhuang Dong, Xiantao Shen, Bin Wu

**Affiliations:** 1 School of Chemistry and Chemical Engineering, Hubei Polytechnic University, Huangshi 435003, China; 2 State Key Laboratory of Environment Health (Incubation), Key Laboratory of Environment and Health, Ministry of Education, Key Laboratory of Environment and Health (Wuhan), Ministry of Environmental Protection, School of Public Health, Tongji Medical College, Huazhong University of Science and Technology, Hangkong Road #13, Wuhan 430030, China; 3 Anheuser-Busch Management (Shanghai) Co., Ltd. Wuhan Branch, Wuhan 430051, China; bin.wu@budweiserapac.com

**Keywords:** yeast, molecularly imprinted polymers, precipitation polymerization, emulsion polymerization, surface polymerization, supporting substrate

## Abstract

Molecularly imprinted polymers (MIPs) have gained significant attention as artificial receptors due to their low cost, mild operating conditions, and excellent selectivity. To optimize the synthesis process and enhance the recognition performance, various support materials for molecular imprinting have been explored as a crucial research direction. Yeast, a biological material, offers advantages such as being green and environmentally friendly, low cost, and easy availability, making it a promising supporting substrate in the molecular imprinting process. We focus on the preparation of different types of MIPs involving yeast and elaborate on the specific roles it plays in each case. Additionally, we discuss the advantages and limitations of yeast in the preparation of MIPs and conclude with the challenges and future development trends of yeast in molecular imprinting research.

## 1. Introduction

Molecular imprinting [1,2,3] is a biomimetic molecular recognition technique that replicates the specificity observed in biological molecule recognition, such as antigen–antibody and enzyme–substrate interactions. So far, molecular imprinting has been widely used in chemical separation [4], disease diagnosis [5], biochemical sensing [6,7,8,9], and other areas involving recognition and detection. The fundamental principle of molecular imprinting involves the preparation of MIPs by polymerizing monomers, crosslinkers, and other essential components (initiators and porogens) in the presence of a target template [10]. Following the template removal, imprinted sites are formed within the polymer matrix, matching the size, spatial structure, and binding sites of the target template [11]. These imprinted sites allow for the selective recognition and binding of the target template, endowing MIPs with the characteristics of artificial receptors that exhibit high affinity and selectivity towards the target molecule [12]. MIPs offer numerous advantages, including excellent mechanical stability, thermal stability, chemical stability, cost-effectiveness, and mild operating conditions. However, there are still some challenges in practical applications of MIPs, such as limited binding affinity to the target molecules, slow mass transfer rates, and difficulties in template removal.

Over the past few years, researchers have focused on addressing these challenges through the exploration of different supporting substrates in molecular imprinting, aiming to optimize the synthesis process of MIPs and enhance their recognition performance. Conventional carriers used in molecular imprinting encompass membranes, silica particles, graphene, as well as other organic and inorganic materials [13,14,15,16]. However, these materials often exhibit limited strength and compatibility with the imprinted polymer layers. Recently, bio-materials have emerged as promising alternatives to traditional imprinting matrices in MIP preparation [17]. Among them, yeast stands out as a bio-material with several advantages over the aforementioned carriers, including cost-effectiveness, easy availability, and a greater number of surface chemical functional groups on cell walls without modification steps. Capitalizing on these inherent advantages, yeast holds great promise as a supporting substrate in the molecular imprinting process. The integration of yeast as a carrier in molecular imprinting has optimized the synthesis process of MIPs and significantly improved their recognition performance, providing a versatile tool for the separation or detection of numerous compounds. Consequently, the use of yeast in molecular imprinting bears substantial research significance for the advancement of MIPs. Therefore, it is extremely necessary to provide a critical review to summarize the development of yeast in molecular imprinting.

Numerous comprehensive reviews published in recent years have thoroughly discussed the application capabilities of different MIPs in the molecular imprinting field [18,19,20,21,22,23,24]. However, none of these reviews has demonstrated the role of yeast in molecular imprinting. In this review, we highlight the application of yeast in molecular imprinting, with a specific focus on the preparation methods of MIPs involving yeast. We discuss the advantages and limitations of yeast in molecular imprinting and conclude with the MIP challenges and future development trends of yeast in molecular imprinting research.

## 2. Research Methodology

This study is an analytical bibliographic review related to the preparation of different types of MIPs involving yeast and elaborates on the specific roles it plays in each case. Data collection was carried out from May 2023 to July 2023, using the following databases: PubMed, Science Direct from Elsevier, Wiley Online Library and Springer-Nature, ACS—American Chemical Society, and Google Scholar, as well as databases of scientific articles and patents “The LENS” and “ORBIT Intelligence”.

The inclusion criteria for this work included original articles exclusive to the yeast and MIPs studied, with the full text available in Portuguese, English, and other languages. Exclusion criteria included abstracts, online sites without scientific sources, incomplete texts, and unrelated and repeated articles.

As for the search strategy, the descriptive words used in this work were as follows: *Molecular imprinting*; *Imprinting Technique*, *Molecular*; *Molecular Imprinting Techniques*; *Techniques, Molecular Imprinting*; *Yeast*; *Fungus*. The articles were selected by reading the titles and abstracts of the publications, associated using the Boolean descriptor “AND”, to refine the samples.

**Figure 1:**
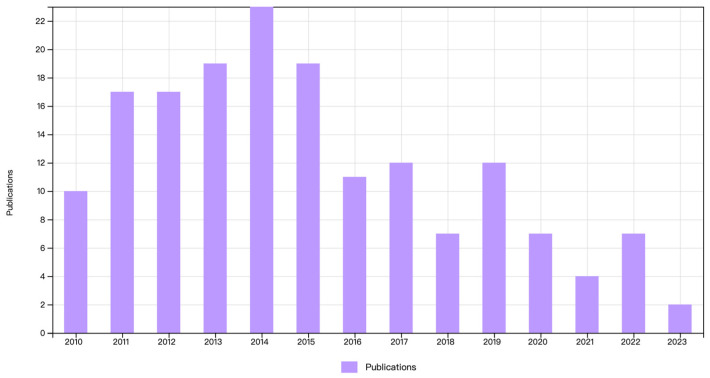
The publishing trend for yeast–MIP articles from 2010 to 2023.

The review is based primarily on articles published after 2010. However, some older articles were also mentioned to provide relevant background or well-documented information. As seen from Figure 1, the research on yeast and MI reached its peak around 2014 and has still been reported in recent years. The study shows the preparation of different types of MIPs involving yeast and the potential of yeast in the development of MI, although the current research is limited.

## 3. Development of Yeast in Molecular Imprinting

**Figure 2:**
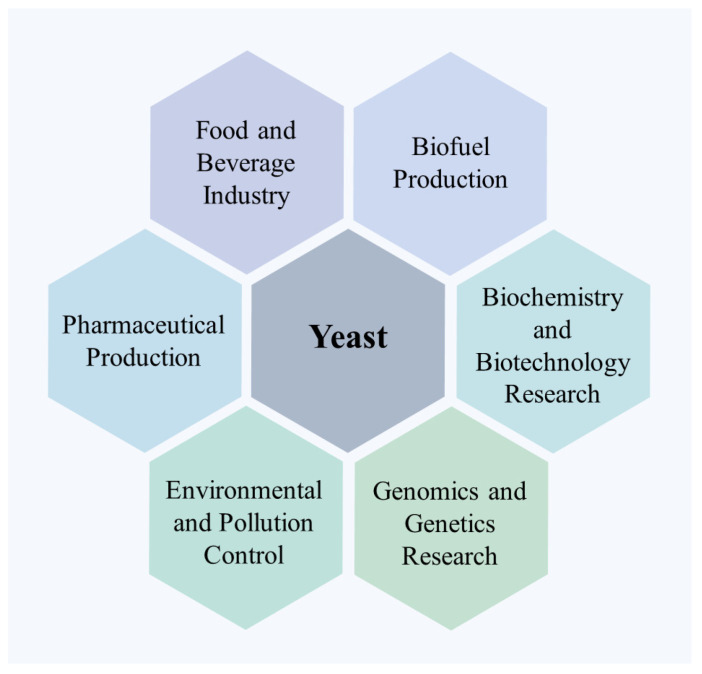
Application areas of yeast.

Yeast, a fungus, is a relatively large eukaryotic and single-celled organism (usually with a diameter of 2.0–4.0 μm). Yeast is widely distributed in nature and possesses advantages such as a short growth cycle, fast reproduction rate, simple structure, easy cultivation, relatively simple genome, and convenience of operation. These advantages make it a widely used model organism for research in the fields of biology and genetics [25,26]. These characteristics of yeast have also prompted researchers to use in a wider range of fields [27,28,29,30,31] (Figure 2), including molecular imprinting.

First and foremost, as the first extensively studied and stable model organism, yeast can be used as a target for studying the mechanisms of molecular imprinting. For example, Derick et al. [32] used yeast as a model cell to investigate factors that influence the recognition of target molecules by MIPs. It is well known that the selective recognition of target molecules by MIPs is partially dependent on the geometric matching between the target molecule and the imprinting cavities on the MIPs. However, the understanding of the binding mechanism is still limited. The authors studied the factors that influence the recognition of surface-imprinted polymers by cells using spectroscopic and microscopic techniques, as well as a heat transfer method-based transducer platform. They demonstrated the significant role of phospholipids in the cell–SIP binding mechanism, which is important for the preparation of more efficient MIPs and the improvement of the detection limit of SIP-based sensors. Secondly, yeast recognition is of significant value in fields such as brewing. For example, Jamieson et al. [33] developed a heat analysis-based sensor that used electro-polymerization to immobilize MIPs on screen-printed carbon electrodes for the precise detection of yeast. In summary, such studies have given a better understanding of yeast’s biological characteristics and molecular imprinting mechanisms and contributed to the development of new biosensors. Initially, researchers used MIPs to detect and recognize yeast specifically. In this process, the identification was usually achieved based on the yeast’s surface characteristics, and thereby the unique merits of yeast, especially its stability, were observed and made it widely used as a supporting substrate. Yeast, as an excellent biomaterial, is being increasingly explored and applied in molecular imprinting due to its excellent material properties.

Over the years, a large number of molecularly imprinted materials (MIMs), including materials at the nanoscale, have been carefully designed and applied in molecular imprinting [34]. Among these materials, core–shell MIPs synthesized with imprinting carriers has gained considerable attention in molecular imprinting. Currently, imprinting carriers such as silica gel, nanotube membranes, and graphene [34,35,36] have been extensively reported. In the case of yeast, its cell wall is rich in active biomolecules, including glucans, mannans, chitin proteins, and a small amount of lipids. It also has many surface chemical groups, such as carboxyl (–COOH), carbonyl (–C=O), amino (–NH_2_), hydroxyl (–OH), and phosphate (–P=O) groups. These characteristics make yeast suitable for molecular imprinting without further modification. Additionally, yeast has the advantages of low cost and ease of cultivation compared to other imprinting carriers such as silica gel, chitosan, nanotube membranes, and graphene. As seen from Table 1, MIPs using yeast as a supporting substrate have been widely used in the detection of antibiotics, pesticides, and other compounds [37,38,39,40,41,42,43].

**Figure 3:**
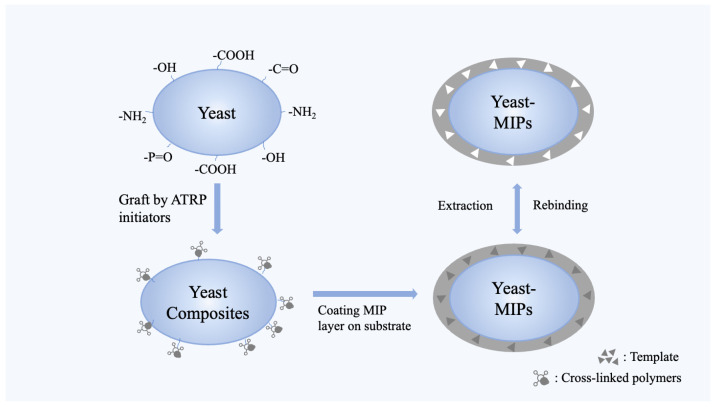
The synthesis of MIPs using yeast as supporting substrate. Adapted from Ref. [37].

The utilization of different materials provides the basis for the separation or detection of numerous compounds, including large molecules and cellular structures, thereby promoting rapid development in the field of molecular imprinting. In 2012, Li et al. [37] described the use of yeast as a carrier in molecular imprinting for the preparation of yeast surface-imprinted polymers using atom transfer radical polymerization to remove cefalexin. Since then, yeast as a support substrate in molecular imprinting has gained attention. The process of MIP preparation involving yeast typically consists of the following steps [44,45]: (1) Binding of template on supporting substrate where the template molecule interacts with the surface of the support substrate. In general, the template molecule is usually a structural analogue of the target molecule. The template molecule can bind to the surface structure of the support substrate through physical adsorption, covalent bonding, or other chemical reactions. Sometimes, some modifications of the support substrate are required in order to bind the template to the supporting substrate. Taking the typical preparation of MIPs using the ATRP principle as an example, the ATRP initiator can be introduced into the yeast surface in advance to avoid adverse reactions such as free radical coupling or disproportionation, facilitating the growth of MIPs on the yeast [37]. (2) Formation of the imprinted polymer where, in the presence of the template molecule, an imprinted polymer layer is formed on the surface of the support substrate through polymerization reactions. Functional units in the polymer interact specifically with the template molecule, thereby forming pore structures in the polymer that are similar in specificity to the structure of the target molecule. It is worth noting that compared to the size of yeast, the molecular imprinting layer is only about 0.5–1.0 microns, and it shows a rough sphere-like structure under the support of yeast. (3) Elution of the template molecule where an appropriate eluent is selected to disrupt the interactions between the template molecule and the functional monomers, allowing the template molecule to be washed out from the polymer (Figure 3). Consequently, the surface of the supporting substrate is engineered to form imprinting cavities that precisely match the size, shape, spatial arrangement, and functional groups of the template molecules, resulting in the creation of MIPs specifically designed for the recognition of the template molecule.

In order to achieve the selective recognition and capture of target molecules, yeast is commonly employed as a supporting substrate in molecular imprinting techniques. The general principle involves a simple pretreatment of the yeast and creating an imprinted polymer layer on its surface. However, the precise role of yeast varies across different methods utilized for synthesizing MIPs.

## 4. Involvement of Yeast in Different Types of MIP Synthesis

According to the interactions between the template molecules and functional monomers, MIPs can be classified into three types: covalent MIPs, non-covalent MIPs, and semi-covalent MIPs [46]. In the synthesis process of MIPs involving yeast, most of the studies used yeast powder which is more convenient than yeast cells. Yeast is usually pre-treated by mixing and drying with normal saline, ethanol, and organic solvents, and also faces different pHs, high temperature, and high pressure. During this process, the yeast particles usually maintain their intact morphological characteristics, and the groups on their surface usually change in order to facilitate the subsequent process of synthesizing polymers on their surface [39,40,41]. Yeast is used as supporting substrate and stabilizer in the synthesis of various types of MIPs, including precipitation polymerization, emulsion polymerization, and surface imprinting. These MIPs have been successfully applied in the detection of antibiotic residues, pesticide residues, and in other fields [40,41,42].

### 4.1. Yeast’s Involvement as a Supporting Substrate in Precipitation Polymerization

Precipitation polymerization is a widely used method for the preparation of spherical particles. The basic principle involves adding reactants including template molecules, functional monomers, and cross-linking agents to a large amount of solvent. Initially, pre-assembly occurs, followed by the addition of an initiator to initiate the polymerization reaction at a specific temperature. After template removal using an elution agent, MIPs with specific imprinting cavities are obtained. This method typically yields uniformly sized spherical particles with a large surface area and significant selective recognition capabilities [47]. Precipitation polymerization is an operationally convenient method with high yields, making it one of the simplest methods for preparing spherical polymers.

In recent years, reversible addition–fragmentation chain transfer (RAFT) [48,49,50] and ATRP [51,52] techniques have been applied to precipitation polymerization methods. By controlling the reaction rate and increasing the polymer particle size, these techniques allow for broader applications. However, strict control of reaction conditions and consideration of the materials used during the experimental process are necessary to obtain spherical particles with strong recognition selectivity and uniform size. Therefore, several factors need to be considered when selecting the precipitation polymerization method.

To address these issues, Ma et al. [42] developed a mild and effective method for preparing thermo-responsive and magnetic MIPs based on magnetic yeast. The researchers modified the yeast surface with Fe_3_O_4_ nanoparticles and used an in situ precipitation polymerization method to prepare thermo-responsive magnetic molecularly imprinted polymers (TMMIPs) on the yeast surface, which was proved by FT-IR spectra: the peaks at 609 cm^−1^ were assigned to Fe–O bond vibrations of Fe_3_O_4_, which demonstrates that Fe_3_O_4_ was successfully introduced to mag-yeast and TMMIPs. The TMMIPs exhibited excellent stability and regenerability. They were successfully applied to the selective adsorption and release of tetracycline from aqueous solutions. The use of yeast as a support substrate offers advantages such as low cost, easy availability, and the presence of abundant active biomolecules on the cell wall, eliminating the need for further modification. More importantly, yeast provides a platform on which Fe_3_O_4_ nanoparticles can be evenly distributed, or else the Fe_3_O_4_ nanoparticles would be messy and could easily agglomerate. This study highlighted the promising role of yeast as a supporting substrate in the molecular imprinting process.

### 4.2. Yeast’s Involvement as External and Internal Supporting Substrates in Emulsion Polymerization

In molecular imprinting, emulsion polymerization is another commonly used technique for preparing MIPs. In this method, the reactants including template molecules, functional monomers, and cross-linking agents are dissolved in a certain amount of organic solvent. The mixed solution is then transferred to an aqueous phase and emulsified by stirring, followed by the addition of an initiator to initiate the polymerization reaction. The MIPs obtained through this method have a controllable and uniform particle size, large surface area, and the ability to imprint water-soluble target analytes [53,54,55]. However, the use of surfactants in this method makes the cleaning process cumbersome, and the particle size of these MIPs is usually small, with a weak compressive strength and poor stability in practical applications.

Common solid stabilizers in Pickering emulsions include inorganic materials such as silica, iron oxide nanoparticles, and calcium carbonate, as well as clays such as laponite and montmorillonite [56,57,58,59,60]. Compared to these materials, microorganisms have advantages such as low cost, easy availability, and the presence of abundant active biomolecules on the cell wall, eliminating the need for further modification processes. Pravit et al. [61] developed a stable water-in-oil Pickering emulsion based on a bacterial chitosan network, which opened up opportunities for the development of environmentally friendly novel interface materials and the use of bacterial cell networks in novel microreactors.

Inspired by the aforementioned studies, the involvement of yeast as external and internal support substrates in emulsion polymerization has been well-reported in multiple studies. Taking advantage of yeast’s non-toxicity and excellent biocompatibility, Zhu et al. [41] utilized modified yeast as a stabilizer dispersed in the aqueous phase to establish stable water-in-oil Pickering emulsions. The magnetic molecularly imprinted microspheres (MMIMs) were acquired through Pickering emulsion polymerization. The yeast particles, as the emulsion stabilizer, were firstly modified by oleic acid, and then the MIPs formed from the inner oil phase, and the yeast particles attached to the outer surface of microsphere. According to the optical photograph of droplets of the yeast-stabilized Pickering emulsion, it was clearly observed that yeast particles were arranged tightly on the surface of emulsion droplets between the oil and water phases, which suggested that the yeast played a partial support role, enabling the production of MIPs with a high mechanical strength. The MIPs were subsequently employed as adsorbents for selectively adsorbing λ-cyhalothrin in aqueous solutions. Notably, the experimental results confirmed that the exposure of microbial yeast from the imprint microspheres facilitated an increase in λ-cyhalothrin adsorption capacity. This effect can be attributed to yeast’s porous structure and excellent adsorption performance, which aligns with its application principles in areas such as organic dye and pharmaceutical adsorption [28,29].

In the emulsion polymerization of MIPs involving yeast, yeast not only functions as a stabilizer but also participates as an internal support substrate. Wang et al. [40] utilized yeast as a support substrate and employed emulsion polymerization via ATRP to prepare surface-imprinted polymers for the selective recognition and removal of cyclopropane carboxylic acid from aqueous solutions. Compared to traditional multistep ATRP polymers, the use of yeast as a matrix enables a simplified one-step synthesis of yeast–Br composites with a lower energy consumption and higher efficiency. Furthermore, the prepared MIPs exhibit remarkable characteristics such as a high adsorption capacity, rapid binding ability, high selectivity, reusability, and satisfactory recovery rates for trace analysis of CIP in real samples using high-performance liquid chromatography. These MIPs take advantage of yeast’s low cost, easy availability, abundant surface functional groups, and favorable morphology and mechanical properties.

### 4.3. Yeast’s Involvement in Surface Polymerization

#### 4.3.1. Yeast’s Involvement in Surface Polymerization through ATRP

With the in-depth research on molecular imprinting, it has been observed that traditional embedding methods suffer from issues such as deep embedding of imprint recognition sites, difficulties in template molecule removal leading to limited effective recognition sites, and slow responses to template molecules. These problems are still challenging to address, and merely changing the polymerization method does not result in a qualitative transformation. Consequently, surface molecular imprinting has emerged and quickly become a new research hotspot [62].

Surface molecular imprinting refers to the utilization of specific carriers (such as silica nanoparticles, graphene, polystyrene spheres, etc.) that undergo polymerization reactions on their surfaces, making it easily accessible to control the distribution of imprint sites on the surfaces. This facilitates the removal of template molecules and subsequent response and detection processes [63]. The MIPs synthesized using this method overcome issues encountered in traditional MIP synthesis methods, such as deep embedding of template molecules and difficulties in removal. These surface MIPs exhibit advantages such as controllable morphology, uniform particle size, adjustable imprint layer thickness, multiple effective recognition sites, high mass transfer efficiency between template molecules and MIPs, and easy template molecule removal [64].

The selection of support substrates with excellent morphology and mechanical properties is crucial for the preparation of surface MIPs. The use of yeast in molecular imprinting has been proven advantageous through multiple research studies. Li et al. [37] reported an effective ATRP process to achieve yeast-based MIPs. They introduced an initiator onto the yeast surface using the ATRP method, enabling direct growth of MIPs on the yeast surface. They successfully combined the imprint polymer extraction method with high-performance liquid chromatography for the analysis of cefalexin in spiked pork and water samples. The kinetic properties of imprinted polymers were well described by the pseudo-second-order kinetic equation, indicating that the chemical process was the rate-limiting step for the adsorption of cefalexin (CFX). The equilibrium data were well fitted by the Freundlich isotherm, and the multimolecular layer adsorption capacity of the imprinted polymers was 34.07 mg g^−1^ at 298 K. Similarly, Pan et al. [39] also utilized yeast as an imprint carrier and employed atom transfer radical polymerization with electron transfer to prepare temperature-responsive MIPs. This allowed for the selective recognition and enrichment separation of cefalexin in solution. The maximum adsorption capacity of the MIPs at 303 K was 59.4 mg g^−1^, and the maximum release proportion for the MIPs at 293 K in water for 24 h was 71.08%. The selective recognition experiments demonstrated a high affinity and selectivity of the MIPs towards CFX over competitive compounds. Yeast-based MIPs have demonstrated potential applications in the fields of selective separation and enrichment.

#### 4.3.2. Yeast’s Involvement in Surface Polymerization Bonded Magnetic Materials

**Figure 4:**
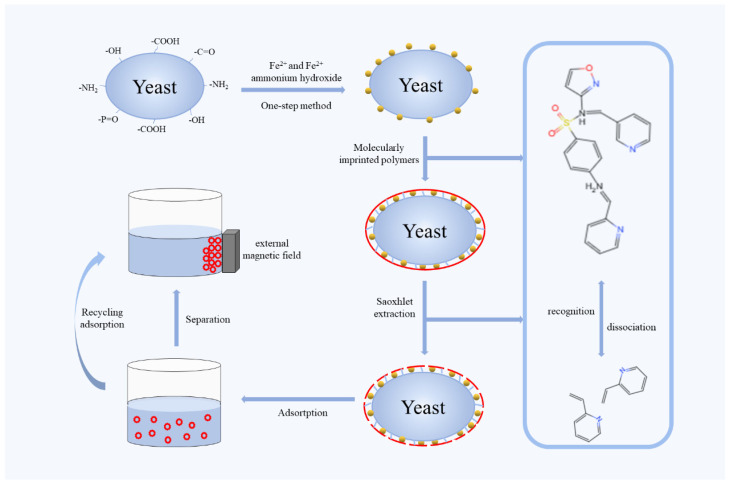
Synthesis approach of magnetic molecularly imprinted polymers. Adapted from Ref. [43].

In addition to using yeast as a standalone solid support for surface molecular imprinting, the inclusion of magnetic materials enables the separation of MIPs after use. Magnetic solid support substrates have the advantage of easy removal from the medium through simple magnetic separation and can be reused. Therefore, researchers have achieved the production of magnetic adsorbents by combining yeast with magnetite (Fe_3_O_4_). Guan et al. [38] developed a method for the efficient synthesis of magnetic surface MIPs using magnetic yeast particles as solid supports by firmly bonding magnetic nanoparticles to yeast through a co-precipitation reaction of FeCl_3_·6H_2_O and FeCl_2_·4H_2_O. This method achieved selective adsorption of trace amounts of β-cypermethrin in contaminated wastewater. The MIPs exhibited an adsorption capacity of 39.64 mg g^−1^ at 298 K. The MIP data fit well in the Langmuir isotherm model compared to the Freundlich model, whereas the kinetic properties of the MMIPs were well described by the pseudo-second-order equation. Qiu et al. [43] used magnetically responsive yeast produced by an in situ one-step method as the support substrate for MIPs that specifically adsorb the antibiotic sulfamethoxazole (Figure 4). The MMIP prepared with magnetic yeast as the support material exhibited a uniform morphology, good magnetization intensity, thermal stability, and excellent specific recognition. Compared to other support materials such as SiO_2_, magnetic yeast particles as carriers did not require intermediate chemical modification steps and significantly improved the grafting efficiency. These findings suggest that surface imprinting polymers based on composite materials combining yeast and magnetite hold great promise as one of the most viable candidates for the separation of environmental pollutants.

In summary, yeast involvement in the synthesis of MIPs offers advantages such as rich chemical functional groups, controllable morphology and structure, biocompatibility, and reusability, among others. These advantages make yeast a promising carrier material with significant research and application value in molecular imprinting.

## 5. Advantages and Limitations of Yeast as Supporting Substrates

The involvement of yeast in MIPs provides a convenient method for producing highly stable and specific MIPs, which can be used for the separation and detection of various molecules. Additionally, the production of composite materials, where the imprinted polymer is combined with yeast as a support substrate and other materials (such as magnetic particles), allows for the removal of environmental pollutants from the medium through simple magnetic separation enabling their recyclability. Various methods have demonstrated the advantages of yeast involvement in MIP synthesis, as seen from Table 2 and is summarized below.

1. Easy availability and low cost: Yeast is easily obtainable and can be cultivated at a low cost in a short time.
2. Rich surface chemical functional groups: The yeast surface contains abundant chemical functional groups, which greatly simplify modification steps and reduce secondary pollution. The presence of hydroxyl, amino, carboxyl, and other functional groups allows for interactions with template molecules, enhancing the recognition and selectivity of MIPs.
3. Controllable morphology and structure: By adjusting cultivation conditions and synthesis parameters, MIPs with controllable morphology and structure can be synthesized on the yeast surface. This tunability enables the design and fabrication of MIPs with specific shapes, pore sizes, and surface properties to meet diverse application requirements.
4. Excellent biocompatibility: Yeast is a natural biological material with good biocompatibility and biodegradability. Using yeast as a carrier for MIPs reduces adverse environmental impacts.
5. Regulation of inorganic material growth: Yeast can regulate the growth of inorganic materials, providing a rich template for the synthesis of nanomaterials through template-assisted synthesis.
6. Diverse microbial cell structures: Microbial cells, including yeast, possess various structures that can serve as templates for the synthesis of nanomaterials, offering a wide range of templates.
7. Addition of magnetic materials: The inclusion of magnetic materials allows for easy separation of the material after use. Magnetic solid supports have the advantage of easy removal from the medium through simple magnetic separation and can be reused.

The application of yeast provides new avenues and possibilities for the development and application of molecular imprinting. However, as a material in molecular imprinting, yeast still has some potential drawbacks such as (1) limitations in reusability. Yeast may suffer damage or deactivation during the molecular imprinting process, limiting its reusability. This could increase the preparation cost and resource consumption of MIMs. (2) A limited applicability range as the chemical and physical properties of yeast may restrict its recognition ability for specific molecules. This limitation narrows the applicability range of yeast as a molecular imprinting material, rendering it ineffective in recognizing certain target molecules.

Despite these potential drawbacks, appropriate optimization and improvement strategies can address these issues to some extent. Moreover, yeast-containing imprint materials show tremendous potential in areas such as adsorption and separation of antibiotics, pesticides, and antibacterial agents [37,41]. This indicates that yeast, as a promising biological material, holds significant research and application value in molecular imprinting.

## 6. Conclusions

This review explored the utilization of yeast in the field of MIPs, with specific attention to the fabrication methods and applications of MIPs utilizing yeast as the supporting substrate. It provided valuable insights into the role and prospects of yeast in the development of molecular imprinting. However, due to the absence of research on the comparative adsorption performance of yeast and traditional substrates using equivalent ratios of template, monomer, and cross-linker compositions under equi-volume and solvent conditions, definitive conclusions regarding the differential adsorption performance between yeast and conventional substrates cannot be established. We firmly believe that future investigations aiming to enhance yeast properties and expand its applications will continue to expand. Prospective research could focus on various domains, especially green strategies, taking into account the numerous advantages offered by yeast as a biomaterial and the increasing emphasis on green chemistry. Exploring more environmentally sustainable research methodologies will be an intriguing challenge.

## Figures and Tables

**Table 1:** Representative literature on yeast involved in MIP synthesis.

Target Molecule	Yeast Type	Type of Polymerization	Polymerization Technique	Imprinting System	Thickness *	Applications	Ref. *
Cefalexin	Yeast powder	Surface imprinting technique	ATRP *	CuCl; MAA *; EGDMA *	0.63 μm	Selective recognition and adsorption of cefalexin	[37]
Beta-cypermethrin	Yeast powder	Surface imprinting technique	None	MAA; EGDMA; M@Y *	1.0–1.1 μm	Selective recognition and separation of beta-cypermethrin from wastewater samples	[38]
Cefalexin	Yeast powder	Surface imprinting technique	AGET ATRP *	Aam *; EGDMA; CuCl2; AsAc *	None	Selective recognition and adsorption of cefalexin	[39]
Ciprofloxacin	Yeast powder	Surface imprinting technique	ATRP	MAA; HEMA *; EGDMA; CuBr	0.365 μm	Selective recognition and removal of Ciprofloxacin from aqueous media	[40]
λ-cyhalothrin	Yeast powder	Pickering emulsions	Thermally initiated radical polymerization	EGDMA; MAA; AIBME *	None	Selective recognition and separation of λ-cyhalothrin	[41]
Tetracycline antibiotics	Yeast powder	Precipitation polymerization	None	MAA; EGDMA; AIBN *	None	Selective adsorption and release of tetracycline from aqueous solution	[42]
Sulfamethoxazole	Yeast cells	Surface imprinting technique	One-step in situ polymerization	AIBN; EGDMA	None	Selective removal of sulfamethoxazole from water	[43]

* Thickness: thickness * of the polymer on the yeast surface; Ref.: reference; ATRP: atom transfer radical polymerization; MAA: methacrylic acid; EGDMA: ethylene glycol dimethacrylate; M@Y: magnetite@yeast composites; AGET ATRP: activator generated by electron transfer atom transfer radical polymerization; AAm: acrylamide; AsAc: ascorbic acid; HEMA: hydroxyethyl methacrylate; AIBME: dimethyl 2,2′-azobis (2-methylpropionate); AIBN: azo-bis-isobutyronitrile.

**Table 2:** Comparison of typical support materials.

Supporting Substrate	Target Molecule	Application	Advantages	Disadvantages	Reference
Silica beads	β-lactamase-resistant penicillins	Determination of beta-lactamase-resistant penicillin residues in complex matrices including milk	The detection limit is much lower than the standard, and the specific recognition effect is good	The preparation process is complex, and the silica is treated in multiple steps	[65]
Chitosan	ketorolac	Determination of ketorolac in human plasma	Non-toxicity, bioavailability, and biocompatibility	—	[66]
Graphene	atropine	Determination of atropine in human serum	Highly selective and sensitive analytical assay for atropine	Interaction between the adjacent graphene sheets or the interaction between the graphene sheets need to be strengthened	[67]
Yeast	cefalexin	Selective recognition and adsorption of cefalexin	Low cost, easily available source, and abundant active biomolecule on the cell wall without further modification process	—	[39]

## Data Availability

Not applicable.
